# 2′-Fucosyllactose modulates gut microbiota and systemic metabolism in mild-to-moderate ulcerative colitis: a randomized crossover trial

**DOI:** 10.1093/ecco-jcc/jjag120

**Published:** 2026-08-24

**Authors:** James M Kennedy, Aminda N De Silva, Patricia Sanz Morales, Carlos G Poveda Turrado, Gemma E Walton, Glenn R Gibson

**Affiliations:** Department of Gastroenterology, Royal Berkshire NHS Foundation Trust, Reading, RG1 5AN, United Kingdom; Food Microbial Sciences Unit, The University of Reading, Reading, RG6 6LA, United Kingdom; Department of Gastroenterology, Royal Berkshire NHS Foundation Trust, Reading, RG1 5AN, United Kingdom; Food Microbial Sciences Unit, The University of Reading, Reading, RG6 6LA, United Kingdom; Food Microbial Sciences Unit, The University of Reading, Reading, RG6 6LA, United Kingdom; Food Microbial Sciences Unit, The University of Reading, Reading, RG6 6LA, United Kingdom; Food Microbial Sciences Unit, The University of Reading, Reading, RG6 6LA, United Kingdom

**Keywords:** prebiotic, ulcerative colitis, inflammatory bowel disease, gut microbiota, clinical trial

## Abstract

**Background and Aims:**

The gut microbiota in active ulcerative colitis (UC) differs from that of healthy individuals. Human milk oligosaccharides (HMOs) are prebiotics found in human breastmilk which promote infant health, but are lesser studied in adults. The objective of this study was to assess the impact of the HMO 2′-fucosyllactose (2′-FL) on symptoms, inflammation, gut microbiota, and systemic metabolism in mild-to-moderate UC.

**Methods:**

In this randomized, double-blind, placebo-controlled crossover clinical trial, 38 patients with mild-to-moderate UC were randomized to receive 28 days of 2′-FL or 28 days of maltodextrin placebo, followed by washout and crossover. Patient-reported outcome measures, feces, urine, and serum samples were collected before and after each intervention, and after a final washout period.

**Results:**

Although no clear treatment effect was observed on conventional clinical indices, 2′-FL exposure was associated with improved patient-reported disease control. 2′-FL significantly modulated the gut microbiota, markedly increasing *Bifidobacterium* abundance (effect size 4.4%, 95% CI 2.33%-6.47%, *P* = 6.5 × 10^−5^). Fecal metabolite profiles showed changes consistent with altered microbial carbohydrate metabolism. Exploratory analyses suggested that age and baseline microbiota may influence clinical and biological responsiveness to 2′-FL.

**Conclusions:**

2′-FL exposure was associated with improvement in patient-reported disease control, gut microbiota modulation, and cross-compartmental host–microbial metabolic profile modifications; Clinicaltrials.gov number, NCT06050811.

## 1. Introduction

Ulcerative colitis (UC) is an inflammatory bowel disease (IBD) defined by chronic inflammation affecting the mucosal layer of the colon, resulting in symptoms of bloody diarrhea, urgency, and abdominal pain. The gut microbiota composition and function in UC is different to that of healthy people, with a reduction in abundance of more beneficial bacteria such as *Bifidobacterium* spp. and *Faecalibacterium prausnitzii*, with a corresponding reduction in beneficial metabolites such as short chain fatty acids (SCFAs) in the colon during active inflammation.^1^^,^^2^

Modulating the gut microbiota in UC to positively impact on inflammation and symptoms has long captured scientific interest, for example via fecal microbiota transplantation, probiotics, or prebiotics. Prebiotics are substrates that are “selectively utilized by host microorganisms conferring a health benefit.”^3^ Although the potential leverage prebiotics have over effecting microbiota change in UC is recognized, high-quality clinical trials of prebiotics in UC are few and far between.^4^

The evidence base for dietary therapies for UC is limited, despite the role of diet in IBD pathogenesis being well established.^5^ Patients' desire to know what dietary changes can influence their disease course is clear.^6^

Human milk oligosaccharides (HMOs) are prebiotics found in human breastmilk which do not directly nourish the infant, but which have various health benefits.^7^^,^^8^ These benefits are exerted primarily via the microbiota, but also via direct pathogen inhibition, immune modulation, reduced inflammation, and host effects following systemic absorption.^9^

In the present study, we sought to study the effect of a prebiotic candidate in mild-to-moderate UC in an adequately powered, randomized, double-blind, placebo-controlled crossover clinical trial. A pre-competitive approach using an *in vitro* batch fermentation model was carried out to select the most promising prebiotic candidate.^10^ The prebiotic potential of the HMO 2′-fucosyllactose (2′-FL), an oligofructose-enriched inulin (fructo-oligosaccharide, or FOS), and a galacto-oligosaccaride (GOS) mixture, were compared with feces from patients with active UC. Although all three prebiotics had a positive effect on the microbiota *in vitro* and SCFA profile, the HMO 2′-FL was found to best suppress *Desulfovibrio* spp. (sulfate-reducing bacteria recognized as being relevant to UC), and significantly increased acetate production over 48 h of fermentation. It was therefore taken forward as the candidate for the human intervention trial.

## 2. Materials and methods

### 2.1. Trial design

The clinical trial followed a randomized double-blind placebo-controlled crossover design. The trial protocol was prospectively registered with ClinicalTrials.gov as Prebiotics in Reducing Inflammation and Clinical Endpoints in Ulcerative Colitis (PRInCE-UC, NCT06050811, registered September 8, 2023). The study was carried out with ethical approval from the United Kingdom National Health Service Health Research Authority, Research Ethics Committee reference 23/NW/0080, and was conducted in line with terms set out by the Declaration of Helsinki (2024).^11^

Based on an expected placebo response rate of 20%, a therapy response rate of 50%, and an intra-subject correlation of 0.5, 19 patients were required to complete both arms of the crossover trial per protocol to detect a difference between 2′-FL and placebo with a significance level of 0.05 and power of 80%. These intervention and placebo response rates are consistent with those used in powering a randomized controlled trial in Crohn’s disease using prebiotic FOS.^12^ The recruitment target was inflated to account for anticipated attrition, including loss to follow-up, protocol deviations, and missing or ineligible biological samples.

Patients were recruited to the trial via the IBD service at the Royal Berkshire NHS Foundation Trust, Reading, UK. They were enrolled according to the below inclusion and exclusion criteria, and, following a screening telephone call and questionnaire, invited to a baseline trial visit. At this visit, stool, urine, and blood samples were taken, and patients completed 24-h dietary recall and baseline symptom questionnaires. Study data were collected and managed using REDCap (Research Electronic Data Capture) electronic data capture tools hosted at the University of Reading.^13^^,^^14^

Symptom questionnaires used on each visit were the Simple Clinical Colitis Activity Index (SCCAI), IBD-Control-8, Bristol Stool Chart, and (4 point) Likert-type scales of flatulence and bloating.^15^^,^^16^

Participants were then randomly assigned to an intervention-then-placebo group or placebo-then-intervention group using Sealed Envelope.^17^ Both participants and investigators were blinded to the product order of each sequence. The intervention used for the trial was 5 g 2′-FL per day (DSM-firmenich, Switzerland) and the placebo was 5 g maltodextrin per day (Kent Foods, UK). Participants then took 28 days of the designated product alongside their usual care. They were instructed to take the product at the same time every day, to log any missed doses or side effects, and to make no significant dietary changes during the trial period.

Following this first treatment period, there was a further visit with questionnaires and biofluid samples, followed by a 21-day washout period during which no product was consumed. There was then a further visit before the second (crossover) treatment period when the participant took the product not taken initially, followed by a final visit after a 21-day washout period to ascertain duration of any potential prebiotic effect and assess for carryover. Figure S1 is a schematic outlining patient flow throughout the trial.

### 2.2. Study subjects and treatment eligibility

Inclusion criteria were: signed consent form; adults (aged from 18 to 64 years); diagnosis of UC by endoscopy and histology; mildly or moderately active UC (based on symptom score and gastroenterologist opinion, with elevated serum C-reactive protein (CRP) above reference range for local laboratory and/or fecal calprotectin of 150 μg/g or greater and/or endoscopic disease activity, the latter three criteria having been within the past 2 months).

Exclusion criteria were: patients with acute severe colitis, as defined by the Truelove and Witts criteria;^18^ intake of any experimental drug within 4 weeks prior to study; former participation in prebiotic or laxative trials within the previous 3 months; use of antibiotics within the previous 4 weeks; introduction of an immunomodulator or advanced therapy (eg, biologic) within 12 weeks or dose change of an immunomodulator or advanced therapy within 6 weeks; introduction of oral 5-ASA within 8 weeks, or dose change of an oral 5-ASA agent within 2 weeks; use of corticosteroids within the preceding 6 weeks or during the trial period; intake of other specific prebiotics (such as oligosaccharides [eg, inulin]), or probiotics (eg, live yoghurts, other fermented products), drugs active on gastrointestinal motility, or a laxative of any class, for 4 weeks prior to the study; women who are lactating, pregnant or planning pregnancy during the study period.

### 2.3. Primary and secondary outcomes

The primary outcome was clinical response as defined by a decrease in SCCAI of greater than or equal to two points from baseline. Secondary endpoints were induction of remission; patient-reported disease control; bowel habit as measured by Bristol stool score; bloating and flatulence; change in fecal calprotectin; changes in fecal microbiota; and serum, urine, and fecal metabolites.

### 2.4. Adverse events

Participants were advised to contact the trial team immediately if adverse events occurred, and additionally these were screened for and recorded at each trial visit in accordance with Good Clinical Practice.

### 2.5. Sample collection, processing, and storage

Participants attended a study visit where questionnaire responses were confirmed, venepuncture for serum was performed, and fecal and urine samples were provided. Participants were instructed to collect both the fecal and urine sample as soon as possible before the study visit. The feces were transported in an airtight container on ice from the participant’s home to the study visit, and then again on ice to the study laboratory where aliquots were promptly frozen at −80 °C. A 10% (w/v) fecal slurry was formed by stomaching an aliquot of the fecal sample with phosphate buffered saline (PBS; 10 mM phosphate, pH 7.4). This was then used downstream for bacterial enumeration and metabolomic analyses.

Urine and blood samples were centrifuged at room temperature at 2000 *g* for 10 min. The supernatant (acellular urine and serum respectively) was then aliquoted into 2-mL cryotubes for storage at −80 °C in anticipation of downstream processing.

### 2.6. Fluorescence *in situ* hybridization flow cytometry for total bacteria enumeration

In total, 750 µL of the fecal slurry was prepared for bacterial enumeration using fluorescence *in situ* hybridization (FISH) combined with flow cytometry (FC) as described by Grimaldi et al.^19^ Sample preparation for FC is detailed in the Supplementary Methods. Unless otherwise stated, all chemicals and reagents were obtained from Sigma (UK).

FC was performed using an Accuri C6 flow cytometer and analyzed using the Accuri CFlow Sampler software (both BD Biosciences, UK). Bacterial counts were calculated using cytometry counts and appropriate dilution factor.

### 2.7. 16S rRNA gene sequencing

Bacterial DNA was extracted from fecal samples using a QIAamp® PowerFecal® Pro DNA Kit (Qiagen, Germany) according to the kit protocol. Extracted DNA quality and quantity were confirmed using Thermo Fisher Scientific Nanodrop 1000 3.8.1. Amplicon generation, library preparation, and 16S rRNA gene amplicon sequencing were performed by Novogene (Cambridge, UK). The V3–V4 region was amplified using barcoded primers 341F and 806R and sequenced on an Illumina NovaSeq 6000 platform to generate paired-end reads. Raw FASTQ files were returned to the investigators and used for all downstream analyses.

### 2.8. Fecal calprotectin assays

Fecal calprotectin was measured using the CALPROLAB™ Calprotectin ELISA (Firefly Scientific Limited, UK). Samples were prepared using the Calpro EasyExtract™ device as per the product literature, then analyzed using the recommended ELISA procedure. Fecal extract samples were diluted 1:100. In total, 100 µL of each standard, control, and diluted sample were added in duplicate wells. Following incubation, washing, and conjugation steps, 100 µL 1 M sodium hydroxide stop solution was added to each well to achieve a fixed incubation period. The optical density (OD) values were then read at 405 nm using a Spark® multimode microplate reader (Tecan, Männedorf, Switzerland).

### 2.9. Metabolomic analysis using ^1^H nuclear magnetic resonance spectroscopy

Fecal, urine, and serum samples for ^1^H nuclear magnetic resonance spectroscopy (^1^H-NMR) were collected, stored, prepared, and analyzed using established protocols.^20^ Sample preparation for ^1^H-NMR is detailed in the Supplementary Methods.

Data acquisition for biofluid samples was performed on a Bruker Avance III 500-MHz NMR instrument running ICON NMR 4.2 under TOPSPIN 3.6. Fecal water and urine experiments were run using a 1D ^1^H-NMR NOESY presaturation pulse sequence, with 2D ^1^H−^1^H J-resolved (J-Res) and ^1^H−^1^H COSY experiments performed on one QC and one randomly selected single sample to improve resolution and resolve feature overlap. Serum samples were run in the same manner except using a 1D CPMG spin-echo sequence instead of NOESY in order to attenuate residual protein NMR signals. Run order was randomized, and a QC mixture was run as every 10th sample.

### 2.10. Statistical analysis of clinical, microbiological, and metabolomic data

Statistical analysis was performed using R (v.4.5.2; R Foundation for Statistical Computing). The per-protocol (PP) population was pre-specified for primary biological analyses because concomitant antibiotics, corticosteroid initiation, and substantial non-adherence could directly alter the gut microbiota, therefore confounding assessment of intervention-related biological effects. Intention-to-treat (ITT) analyses were performed as sensitivity analyses for clinical outcomes.

Microbiota differential abundance analysis was performed using linear mixed-effects models to account for the crossover design. Models included fixed effects for treatment, phase, and their interaction, with a random intercept for participant to capture within-subject correlation. Estimated marginal means and contrasts were computed to evaluate treatment effects of 2′-FL vs placebo, within-treatment changes, and difference-in-differences. Community diversity analyses were conducted using standard ecological metrics.

For metabolomic analysis, raw Bruker spectra were imported and pre-processed using standard NMR workflows, including baseline correction (asymmetric least squares), chemical shift referencing to TSP, water region exclusion, and peak alignment using parametric time warping. Spectra were binned at 0.01 ppm resolution and subjected to quality-control drift correction using pooled QC samples, with features exhibiting relative standard deviation >30% excluded. Urine spectra were normalized using probabilistic quotient normalization to account for high dilution variability, and fecal and serum spectra using total sum normalization. Metabolites were quantified using automated spectral fitting, with SCFAs additionally quantified by targeted integration over predefined chemical-shift windows. False discovery rate (FDR) correction was applied where relevant to microbiota contrasts and metabolite correlations using the Benjamni–Hochberg procedure.

### 2.11. Statistical analysis for food recall data

Data from the baseline and post-trial (week 14) 24-h food recall questionnaires were collated using Nutritics.^21^ Comparisons of macronutrient and fiber intake were made using paired t-tests for normally distributed data and Wilcoxon signed-rank tests (for non-normally distributed data) to assess any major dietary changes during the course of the study. The ITT population was used for this analysis. All authors had access to the study data and reviewed and approved the final manuscript.

## 3. Results

### 3.1. Study population and trial conduct

**Figure 1.**
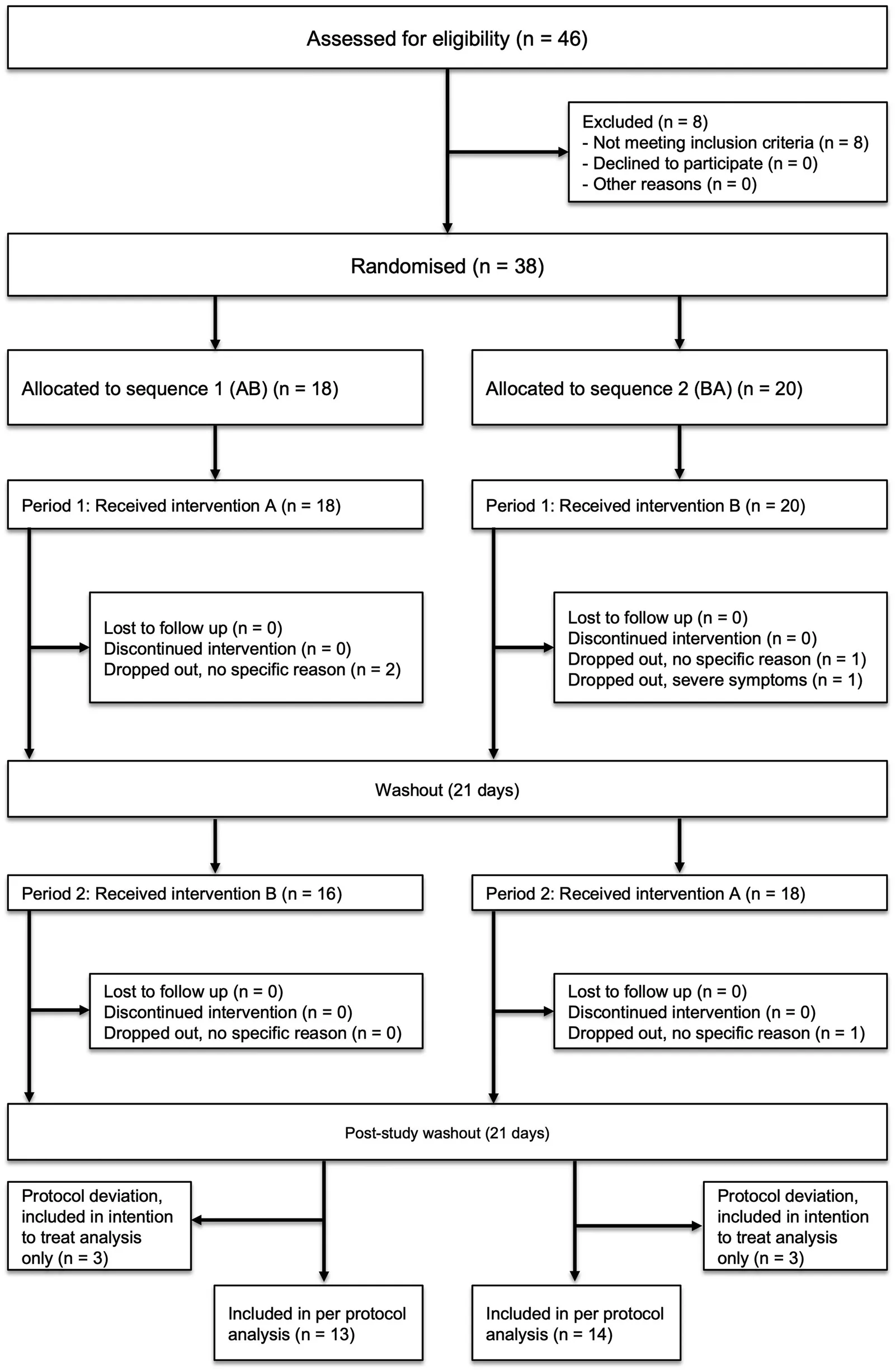
CONSORT flow diagram for PRInCE-UC, a randomized, double-blind placebo-controlled crossover trial of the effects of the human milk oligosaccharide 2′-fucosyllactose (2′-FL) in patients with mild-to-moderate ulcerative colitis.

In total, 38 patients were recruited between January 2024 and February 2025. Thirty-three patients completed the trial (ITT), of whom 27 of these completed the trial according to protocol (PP). Baseline demographic and clinical characteristics were balanced between treatment sequences, and protocol deviations were accounted for through PP analysis. Figure 1 shows the CONSORT flow diagram, and recruited patient characteristics are presented in Table 1.

**Table 1.** Recruited participant characteristics for PRInCE-UC, a randomized, double-blind placebo-controlled crossover trial of the effects of the human milk oligosaccharide 2′-fucosyllactose (2′-FL) in patients with mild-to-moderate ulcerative colitis. Intervention A was 5 g/day maltodextrin placebo, while intervention B was 5 g/day 2′-FL.

		Randomization sequence		*P*
		A then B	B then A	
***N***		18	20	
**Age, years (mean, 95% CI)**		49.9 (44.3-55.6)	44.5 (39.8-49.2)	.129
**Female, % (*n*)**		55.6% (10)	60.0% (12)	.782
**Disease extent, % (*n*)**	E1	33.3% (6)	25.0% (5)	.36
	E2	55.6% (10)	45.0% (9)	
	E3	11.1% (2)	30.0% (6)	
**On immunomodulators, % (*n*)**		27.8% (5)	25.0% (5)	1
**On advanced therapy, % (*n*)**		16.7% (3)	20.0% (4)	1
**On oral mesalazine, % (*n*)**		83.3% (15)	70.0% (14)	.45
**SCCAI at visit 1 (mean, 95% CI)**		5.1 (4.2-6.0)	4.7 (3.6-5.7)	.482
**IBD-Control-8 at visit 1 (mean, 95% CI)**		12.5 (11.2-13.8)	11.3 (9.7-13.0)	.266
**Calprotectin, μg/g (median, IQR)**		244.3 (54.6-435.1)	265.0 (190.9-519.9)	.317

Adherence to both products was high (>85%, see Supplementary Methods for details) and comparable between interventions. Five participants discontinued the study; this occurred during both intervention periods (three during maltodextrin [MDX] and two during 2′-FL exposure). Four of these discontinuations were patient choice unrelated to the intervention, and one was due to progression to severe UC requiring escalation of treatment.

2′-FL was very well tolerated, with no serious adverse events and no differential adverse event frequency between intervention periods. Reported gastrointestinal symptoms were mild to moderate and occurred during both intervention periods. No evidence of carryover effects was observed; formal assessment of carryover effects and crossover performance is presented in the Supplementary Results.

The 24-h dietary recall demonstrated no significant difference between participants' baseline macronutrient and fiber intake and that recorded at the end of the trial (see Supplementary Results).

### 3.2. Clinical and patient-reported outcomes

No significant difference was seen in the primary outcome of clinical response (decrease in SCCAI by *≥*2) from pre- to post-intervention (2′-FL vs MDX, 18.5% vs 29.6%, *P* = .11), nor clinical remission (SCCAI* ≤ *3) rates (2′-FL vs MDX, 25.9% vs 18.5%, *P* = .32) in the per-protocol cohort.

**Figure 2.**
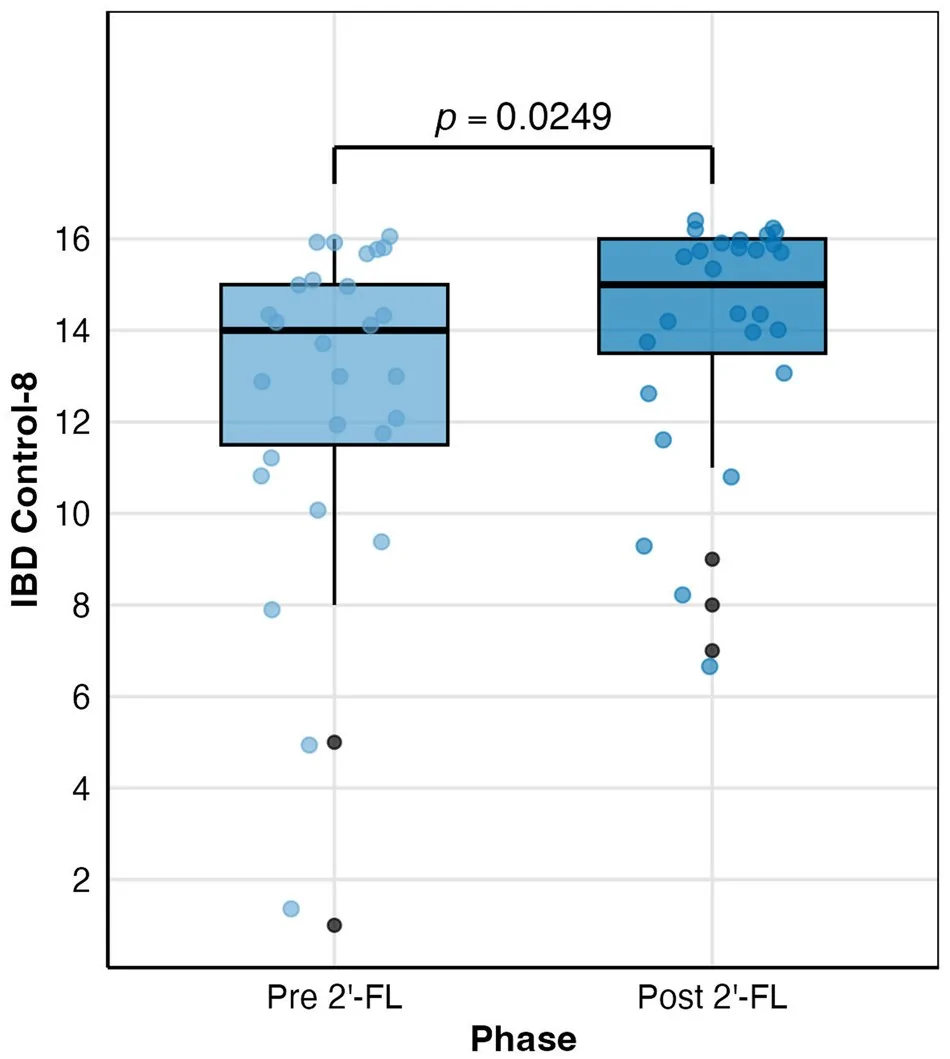
IBD-Control-8 scores before and after 2′-fucosyllactose (2′-FL) supplementation in the per-protocol population, with higher values demonstrating better patient-reported disease control; maximum score 16.

Mean SCCAI scores did not differ significantly between interventions with 2′-FL and MDX (mean post 2′-FL SCCAI 3.74 vs 4.07 with MDX, *P* = .36). IBD-Control-8 scores improved from pre-to-post during 2′-FL supplementation (1.44 point increase, 95% confidence interval [CI] 0.19-2.70, *P* = .025, Figure 2), although no significant treatment effect vs placebo was seen. The clinical relevance of this change should be interpreted cautiously in the absence of a validated minimal clinically important difference for IBD-Control-8, and in the context of no significant change in SCCAI. In addition, although the direction of effect remains consistent, within-arm contrast was reduced in the ITT analysis and no longer remained significant (1.06 point increase, 95% CI −0.13 to 2.25, *P* = .081).

**Figure 3.**
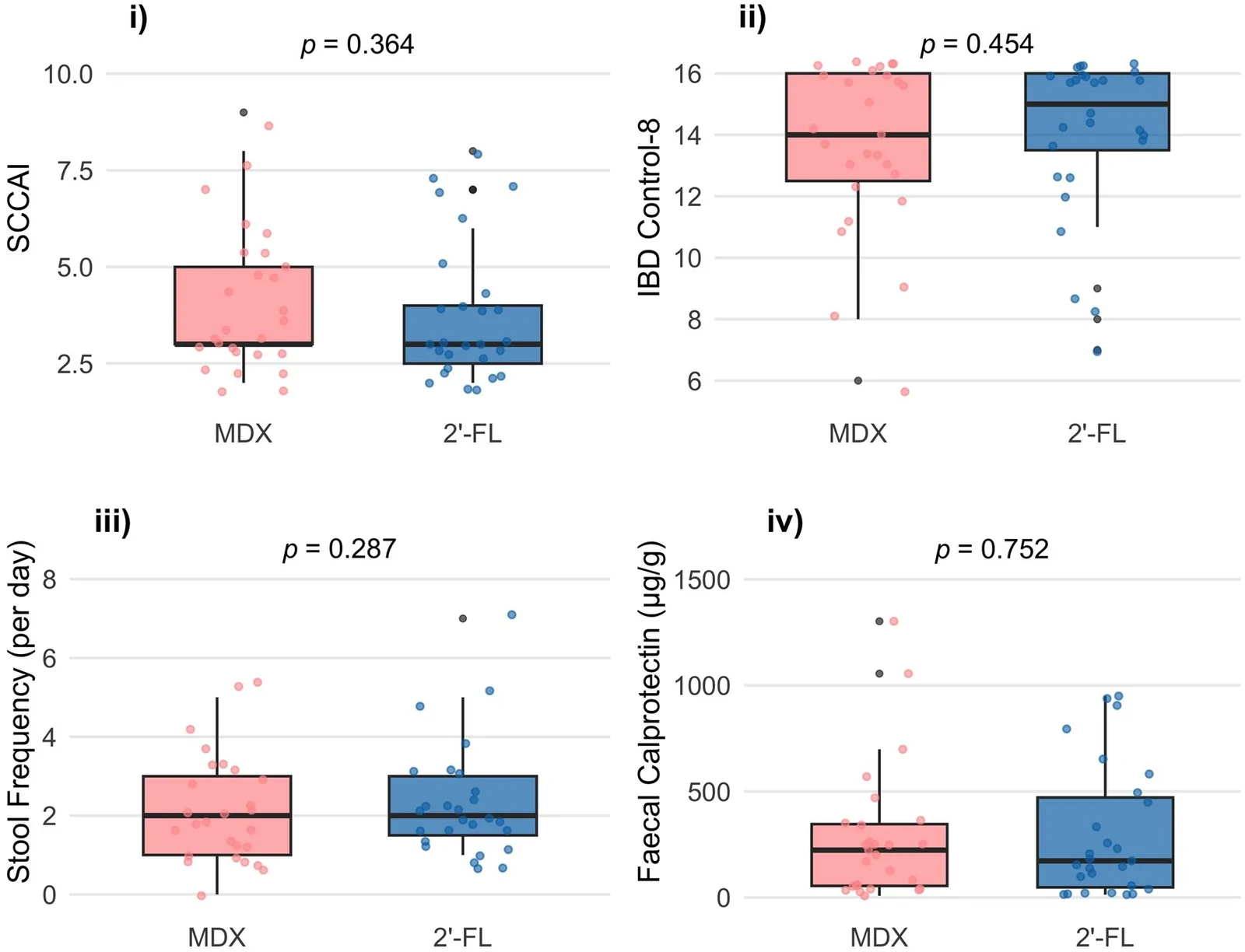
Effect of 2′-fucosyllactose (2′-FL) on (i) Simple Clinical Colitis Activity Index (SCCAI), (ii) IBD-Control-8, (iii) stool frequency, and (iv) fecal calprotectin vs maltodextrin placebo.

A non-significant decrease in mean fecal calprotectin from 342 to 296 μg/g was seen with 2′-FL vs a non-significant increase from 269 to 280 μg/g with MDX. Figure 3 summarizes the key clinical parameter findings. There were no significant differences in any clinical parameter between 2′-FL and MDX in the ITT sensitivity analysis.

### 3.3. Microbiota composition

**Figure 4.**
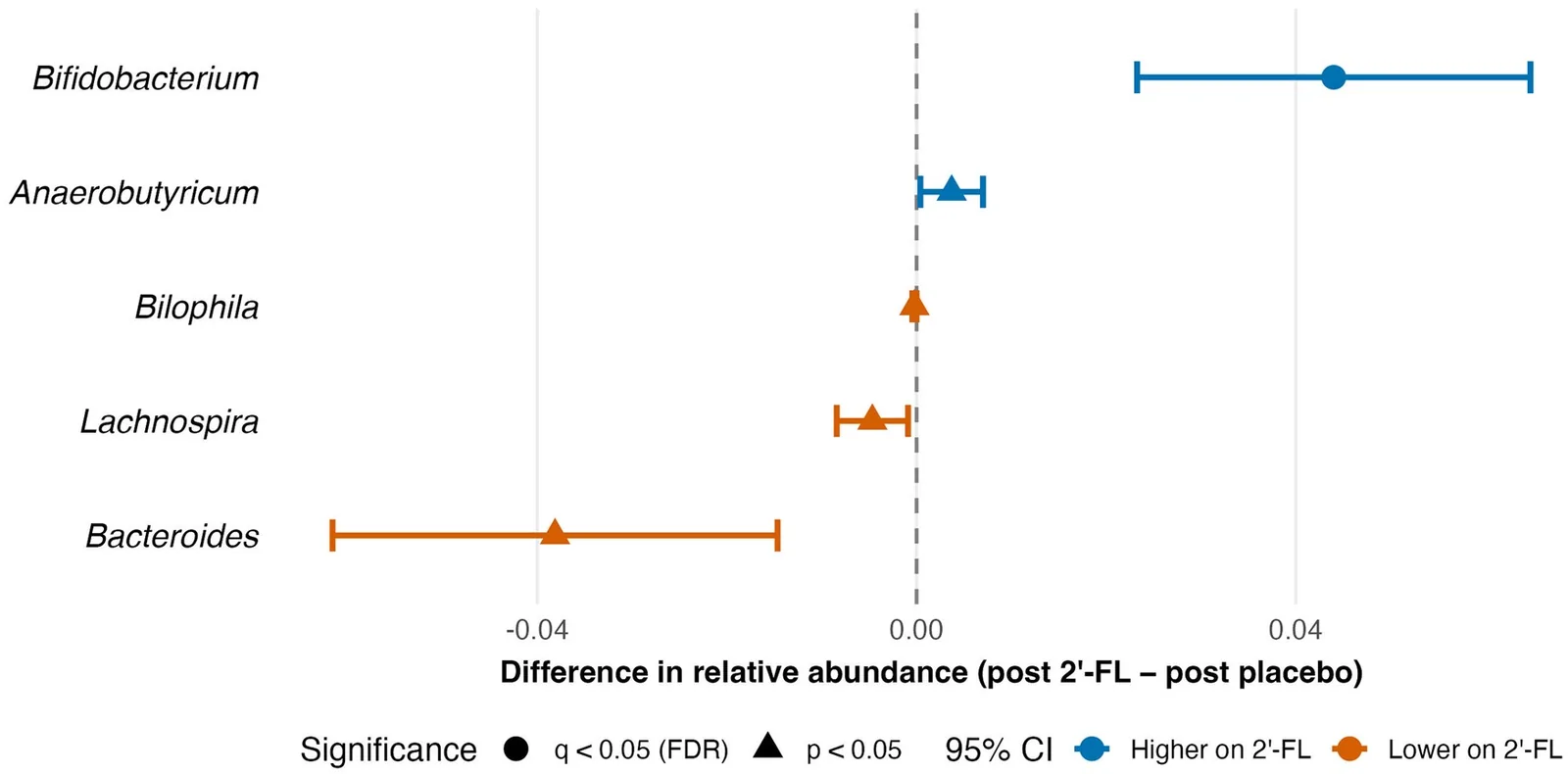
Selected genera showing post-treatment 2′-fucosyllactose (2′-FL) *vs* placebo effect (per-protocol crossover linear mixed model). Circles: false discovery rate *q < *0.05 across all tested genera; triangles: nominal *P* < .05 only.

2′-FL significantly modulated the gut microbiota compared with MDX as measured by both 16S sequencing and FISH-FC (Figure S2). At the genus level, the most marked changes were a significant increase in *Bifidobacterium* with 2′-FL vs MDX (effect size 4.4%, 95% CI 2.33%-6.47%, *P* = 6.5 × 10^−5^), which remained significant following FDR correction (*q *= 0.015), and a decrease in *Bacteroides* (effect size −3.81%, 95% CI −6.16% to −5.1%, *P* = .0018, nominal only; Figure 4). A nominal increase in *Faecalibacterium* (effect size 2.7%, 95% CI 0.096%-5.4%, *P* = .042) was also observed within the 2′-FL arm.

Neither α-diversity nor β-diversity differed significantly with 2′-FL treatment, indicating it did not alter overall microbial diversity nor community structure, respectively, but instead induced specific taxon-level changes in keeping with a prebiotic mechanism.

Cohorting by functional guilds, a significant increase in typical lactate producers (*Streptococcus*, *Lactococcus*, *Lactobacillus*, *Enterococcus*, and *Bifidobacterium*) was seen with 2′-FL vs MDX (relative abundance increase 5.7%-10.7%, *P* = .017, Figure S3).

Overall, 2′-FL selectively enriched saccharolytic taxa, with the dominant microbiota response being an increase in *Bifidobacterium* spp.

### 3.4. Metabolic profiles and cross-biofluid integration

Global metabolic profiles assessed by principal component analysis (PCA) did not differ between interventions in fecal, urine, or serum samples, indicating no strong co-ordinated metabolic shift in any of the three biofluids.

**Figure 5.**
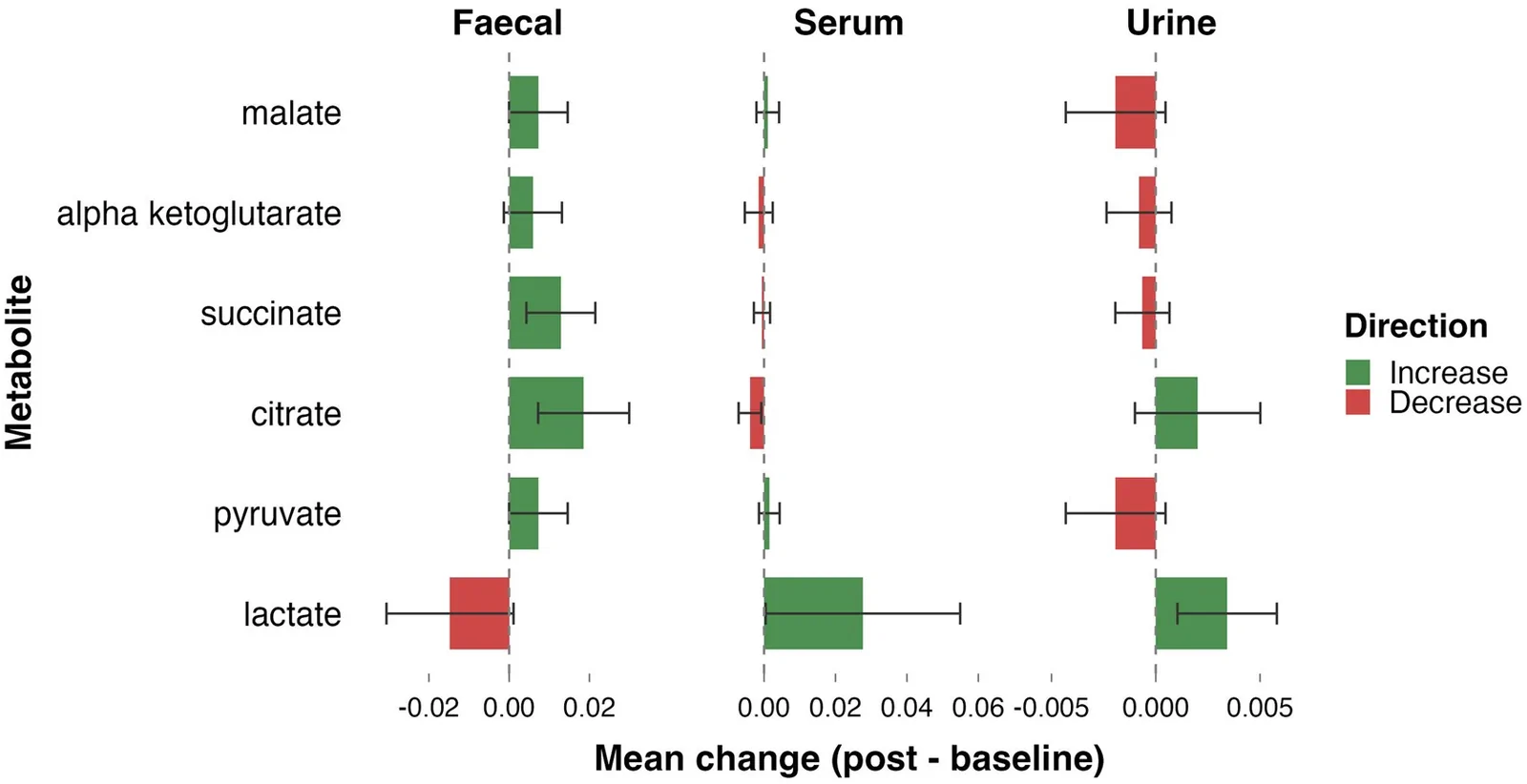
Cross-biofluid changes in lactate and tricarboxylic acid cycle metabolites with 2′-fucosyllactose (2′-FL) supplementation in mild-to-moderate ulcerative colitis.

Targeted analysis of particular metabolites revealed within-period changes during 2′-FL exposure. Directional changes in fecal metabolites were observed during 2′-FL exposure, including increased tricarboxylic acid cycle (TCA) intermediates and decreased lactate (Figure 5).

Directionally consistent but non-significant trends in SCFAs were observed across biofluids. An increase in urinary acetate and butyrate with 2′-FL exposure, along with an accompanying decrease in fecal acetate, indicates co-ordinated microbial-derived metabolites across intestinal and systemic compartments (Figures S4 and S5).

### 3.5. Cross-omic analysis

Clinical, microbiota, and metabolomic data were integrated to explore relationships between microbial taxa and host-microbial metabolites. Across 7920 tested associations, 508 correlations remained significant at FDR < 0.1 and 245 at FDR < 0.05, indicating a structured pattern of microbiota–metabolite relationships (Figure S6).

Microbiota–metabolite correlations showed multiple significant biologically consistent correlations aligning with known microbial metabolic functions. SCFAs correlated positively with known producer taxa, and lactate correlated positively with known producers and negatively with known consumers. Additional correlations were observed across amino acids, TCA intermediates and choline-derived metabolites, which were consistent with known microbial metabolic pathways.

PICRUSt2-predicted functional pathways aligned with measured metabolite changes, further supporting the biologic plausibility of microbiota-associated metabolic changes.

Among exploratory clinical–metabolite analyses, urinary butyrate demonstrated one of the strongest associations with symptom severity during exposure to 2′-FL, with a moderate inverse correlation with SCCAI (*r* = −0.538, *P* = .0038, *q *= 0.038, Figure S7).

### 3.6. Responder analysis

Exploratory responder analyses were performed to identify baseline factors associated with response to 2′-FL. Responders tended to be older than non-responders, with an area under the receiver operating characteristic curve of 0.773, and also demonstrated differing baseline microbiota (Figures S8 and S9). Additionally, higher baseline disease activity was associated with response.

**Figure 6.**
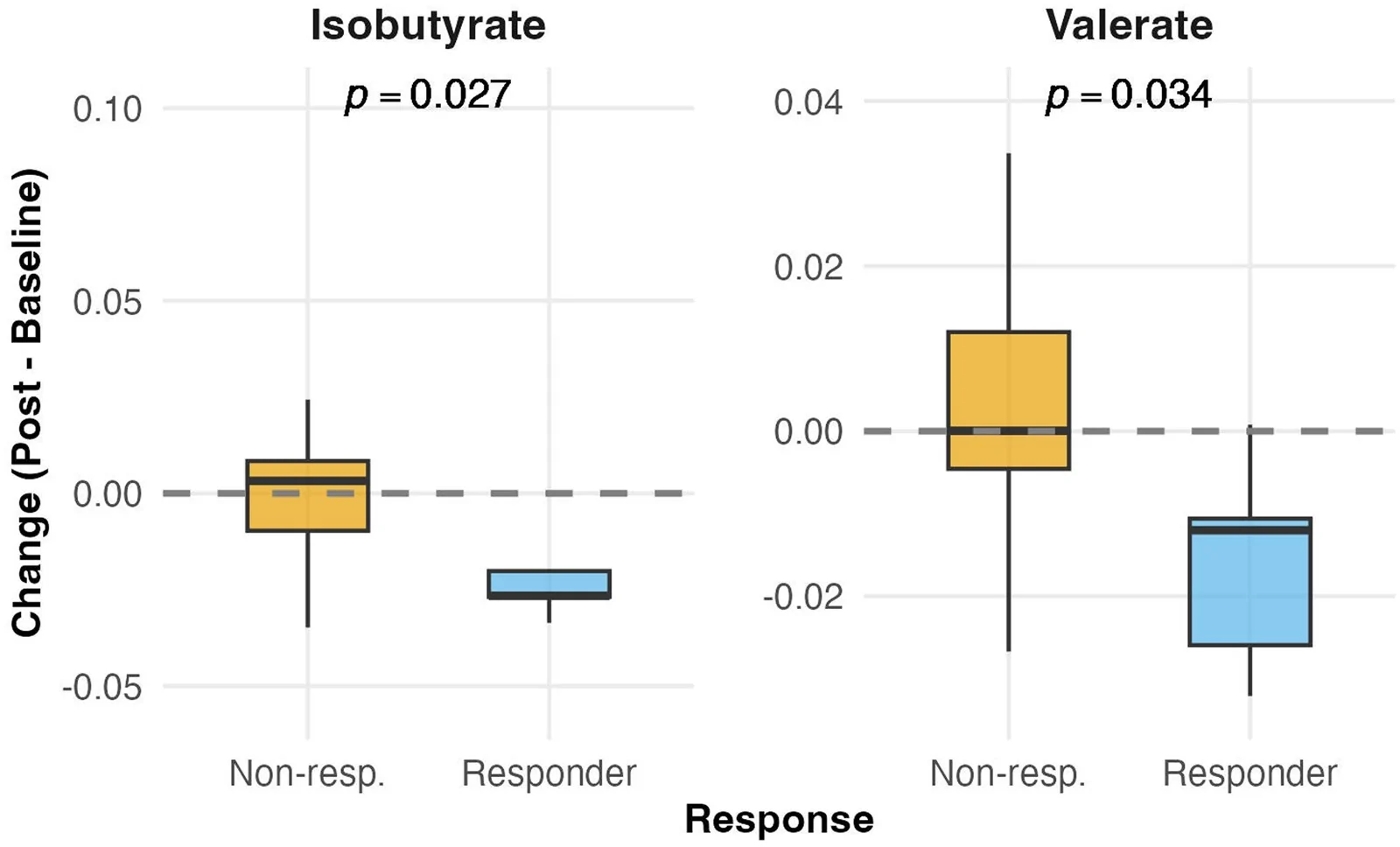
Fecal isobutyrate and valerate changes in Simple Clinical Colitis Activity Index (SCCAI)-defined responders vs non-responders during 2′-fucosyllactose (2′-FL) (per-protocol). Box plots show within-person change in fecal short-chain fatty acid (SCFA) abundance (post-baseline) during the 2′-FL period, stratified by clinical response. Responders were defined as participants with ≥2-point reduction in SCCAI. Dashed line indicates no change.

Clinical responders to 2′-FL exhibited a different SCFA profile to non-responders with lower isobutyrate and valerate, suggesting a shift from proteolytic to saccharolytic activity (Figure 6). Reduced baseline *Bifidobacterium* abundance was associated with 2′-FL response (9.5-fold lower abundance at baseline in responders, *P* = .046), and higher baseline abundance of *Bacteroides*. Given the small number of responders, these findings should be considered exploratory and hypothesis-generating.

## 4. Discussion

This is the first randomized clinical trial of an HMO in UC, and the largest crossover trial of a prebiotic in IBD to date. While no clear treatment effect was observed on conventional clinical indices, 2′-FL exposure was associated with improvements in patient-reported disease control. Marked, biologically coherent gut microbiota modulation and cross-compartmental host–microbial metabolic profile modification was observed with 2′-FL, most strikingly a significant increase in *Bifidobacterium* spp.

*Bifidobacterium* are the most abundant species in the infant gut microbiota.^22^ A higher abundance of *Bifidobacterium* is associated with lower incidence of atopic, autoimmune, and infectious diseases in infants, and they have been shown to have a key immunoregulatory role in early life.^23–26^ Through carbohydrate metabolism, *Bifidobacterium* produce lactate and acetate, which act as substrates for butyrate-producing species such as *Faecalibacterium prausnitzii.*^27^ Although able to metabolize a range of carbohydrates, *Bifidobacterium* species show a predilection for oligosaccharides, in particular fucosylated HMOs such as 2′-FL.^28^

Additional noteworthy microbiota changes in this study include the increase in *Faecalibacterium* seen with 2′-FL. This taxon in particular is a canonical butyrate producer, a reduction in which has been identified as a key signature of UC-related dysbiosis.^29^ Reversing this decrease is a plausible therapeutic target to redress microbiota perturbations associated with inflammation.

SCFAs produced in the colon by the gut microbiota are rapidly processed by the host, for example through absorption into the portal circulation, or colonocyte utilization in the case of butyrate. Measuring metabolites in feces, serum, and urine allowed for cross-compartmental analysis, capturing the pathway from metabolite production in the colon to systemic absorption and renal excretion.

The cross-biofluid SCFA trends seen in this study are consistent with microbiota-associated acetate production with 2′-FL (probably predominantly by *Bifidobacterium* species), with potential downstream cross-feeding of butyrate-producing taxa and higher systemic butyrate. These microbiota-derived metabolic changes are biologically relevant in IBD, and of potential clinical importance. Butyrate is the primary fuel source for colonocytes, and has been shown to be anti-inflammatory and anti-oncogenic.^30^ In particular, butyrate supplementation has been shown to decrease fecal calprotectin and serum CRP in UC, supporting the signal seen in this study.^31^

Microbiota science has evolved sufficiently for us to recognize that rarely do interventions result in simple “cause and effect.” The complex ecosystem of the gut microbiota is a delicate balance of microbe–microbe and microbe–host interactions, meaning that the effect of any given intervention is rarely fully predictable. The question of a divide between responders and non-responders to HMOs has been raised previously, and attributed variously to baseline gut microbiota composition and maternal secretor status.^7^^,^^32^ Exploratory responder analysis suggested heterogeneity in response to 2′-FL, with responders tending to be older, having increased disease activity, and having significantly different baseline gut microbiota. An age-related depletion in beneficial bacteria might explain older patients being more responsive to 2′-FL supplementation, and a higher baseline disease activity might represent a more disrupted gut microbiota more effectively influenced by a prebiotic. The suggestion of greater saccharolytic activity in responders would fit with a prebiotic effect leading to greater carbohydrate metabolism and SCFA production, resulting in benefit to this cohort of patients with UC.

The strengths of this study lie in its crossover trial design and multi-dimensional cross-omic analysis. This study has established a framework for a robust, well-powered dietary intervention study in mild-to-moderate UC, with 2′-FL demonstrating prebiotic potential in this population. The crossover nature of a human intervention trial of microbiota modulating therapy is important; the inter-individual variation in the gut microbiota is so marked that the true value of such a study is derived from observing intra-individual effects.

The pre-competitive methodology whereby provisional prebiotic candidates are first screened in an *in vitro* fermentation model allows for a more efficient and targeted approach to running a prebiotic clinical trial. The validity of this approach was borne out in this study as a directionally concordant bifidogenic effect was seen with 2′-FL in both the *in vitro* and clinical trial results.

Intervention trials with an integrated, multi-omic approach are uncommon in IBD, but offer a functional perspective on how a given intervention interacts with the gut microbiota and host metabolism. Integrating the microbiota, metabolomic, and clinical data allows for a functional rather than just purely ecological view of the effect of a given prebiotic. In addition, the strong microbiota–metabolite correlations seen strengthen the conclusions that any metabolomic changes induced in the host as a result of the intervention were predominantly microbiota-driven.

Limitations of this trial include those to which any real-world dietary intervention trial is susceptible. Although it was demonstrated that participants did not significantly alter their diet during the course of the study, inter-individual variation in diet was large. The intervention duration was clearly sufficient to see some microbiota-related impact, but as these changes may precede clinical benefit the relatively short intervention period may limit assessment of sustained clinical benefit. As the study was powered to an effect size of 30%, more subtle clinical improvements may have been missed. Responder analyses were exploratory and hypothesis-generating only, owing to the relatively small number of clinical responders. In addition, baseline IBD-Control-8 scores were relatively high, indicating that many participants perceived their disease control to be good despite evidence of active inflammation. This may have limited the scope for detecting substantial improvements in patient-reported disease control.

Although 2′-FL alone at the dosage used in this study may not have resulted in clinical response for all participants, the potential role of the many other HMOs in IBD, alone or in combination, has yet to be explored. In this respect, the story of prebiotics in IBD has only really just begun, and will be a promising area of research going forward for clinicians and patients alike.

Clinical effects of a prebiotic are, of course, not expected to match those of immunosuppressive therapy, as we are considering foods as opposed to pharmaceuticals. However, they may represent a low-risk, well-tolerated adjunct to current therapy options to improve disease control and quality of life for people living with UC.

## Supplementary Material

jjag120_Supplementary_Data

## Data Availability

The data that support the findings of this study are openly available in the University of Reading Research Data Archive at https://doi.org/10.17864/1947.001500, reference number 1500.
