# Supplementary material for: 2′-Fucosyllactose modulates gut microbiota and systemic metabolism in mild-to-moderate ulcerative colitis: a randomized crossover trial
Source: J Crohns Colitis. 2026 Aug 24;20(8):jjag120. doi: 10.1093/ecco-jcc/jjag120 (PMC13501536; doi:10.1093/ecco-jcc/jjag120)
Supplement: Supplementary file 1 [file jjag120_supplementary_data.docx]

**Supplementary Methods**

*Sample preparation for FISH-FC*

750 µL of sample was centrifuged at 11 600 × *g* for 5 minutes, and the supernatant discarded. The pellet was resuspended in 375 µL filter-sterilised PBS and fixed in 1125 µL 4% paraformaldehyde, followed by incubation at 4°C for 4 hours to achieve permeabilisation. Samples were then washed twice with filter-sterilised PBS, and resuspended in a mixture of 300 µL filter-sterilised PBS and 300 µL filtered 100% (v/v) ethanol.

25 µL of the suspension was then taken and diluted further in 500 µL filter-sterilised PBS. Samples were centrifuged at 11 600 × g for 3 min, and the pellet was suspended in 100 µL of solution containing 0.1 M Tris/HCl (pH 8.0), 0.05 M EDTA (pH 8.0), and 1 mg ml^−1^ lysozyme. This was incubated in the dark at room temperature for 10 minutes then re-centrifuged and washed with filter-sterilised PBS. The pellet was then suspended in hybridisation buffer (containing 5 M sodium chloride, 1 M Tris/HCl (pH 8.0), 10% sodium dodecyl sulphate, and 30% formamide) and 50 µL aliquoted into Eppendorf tubes, to which 4 µL of the Eub338 I-II-III total bacteria probe (an equimolar combination of Eub338 I, Eub338 II, and Eub338 III) or 4 µL of the negative control Non Eub complementary probe to Eub338 were added (all probes Eurofins Genomics, UK) [1-3].The working solution was incubated at 35°C in a heating block overnight.

Following incubation, the samples were centrifuged and washed using washing buffer (comprising 5 M sodium chloride, 1 M Tris/HCl (pH 8.0), 0.5 M EDTA (pH 8.0), and 10% sodium dodecyl sulphate), then centrifuged again at 11 600 × *g* for 3 min and suspended in 300 µL filter-sterilised PBS.

*Sample preparation for ^1^H-NMR*

Urine and faecal water samples were prepared as follows. 500 µL of the sample was aliquoted into a 1.5 mL Eppendorf tube and centrifuged at 11 600 × *g* for 10 minutes at 4 °C. Internal standard solution was prepared using 0.112 g 3-(Trimethylsilyl)-1-propanesulfonic acid-d6 sodium salt (TSP), 0.1 g 99% sodium azide, and 100 mL deuterium oxide (D_2_O), and then pH adjusted to pH 7 (all reagents Merck, UK). 200 µL of the internal standard solution and 400 µL of the sample supernatant were mixed for 10 seconds using a vortex, and 550 µL of the prepared sample was transferred to a Wilmad® 5mm NMR tube (Merck, UK). Quality control (QC) samples were prepared to contain 50 µL of each sample, then prepared in the same manner.

Serum samples were prepared for ^1^H-NMR as follows: 250 µL of the sample was aliquoted into a 1.5 mL Eppendorf tube and centrifuged at 11 600 × *g* for 10 minutes at 4 °C. A saline solution was prepared using 80 mL High-Performance Liquid Chromatography grade water, 20 mL D_2_O, 0.02 g 99% sodium azide (all reagents Merck, UK). 400 µL of the saline extender solution and 200 µL of the sample supernatant were mixed for 10 seconds using a vortex, and 500 µL of the prepared sample was transferred to a Wilmad® 5mm NMR tube (Merck, UK). Quality control (QC) samples were prepared to contain 40 µL of each sample, then prepared in the same manner.

**Supplementary Results**

*Study population and trial conduct*

With respect to inclusion criteria, eight participants had elevated C-reactive protein, 33 had a faecal calprotectin 150 μg/g or greater, and three had endoscopic disease activity (all mild inflammation) within the previous two months.

Adherence was self-reported by participants and recorded at each post-product visit (i.e. visits two and four). Using an 80% minimum adherence rate, i.e. missing no more than five doses of product out of 28, adherence to both products in the PP group was 100%. In the whole trial population, adherence to MDX was 88.9% (32/36) vs adherence to 2’-FL being 94.4% (34/36) (*p* = 0.67). The dropout rate during MDX was 8.3% (3/36) and during 2’-FL was 5.6% (2/36) (*p* = 1). Median sachet consumption was 28/28 days (IQR 28-28) for both MDX and 2′-FL. Mean consumption was 26.3/28 days (SD 4.9) for MDX and 27.4/28 days (SD 2.0) for 2′-FL; this difference was not statistically significant (Wilcoxon signed-rank *p* = 0.44, n = 34 paired participants).

There were four reported adverse events. With placebo (MDX), three adverse events were reported: one patient developed dry eyes (mild, unrelated to product), one patient developed nausea (mild, possibly related to product), and one patient developed constipation (mild, possibly related to product). With 2’-FL, one patient developed nausea and vomiting (moderate, unrelated to product).

Protocol deviations occurred in six patients. Three patients started steroids during the course of the trial (one during 2’-FL treatment, one during MDX treatment, one during the washout period), two patients took antibiotics during the trial, and one patient took under the 80% adherence threshold of the placebo.

Baseline dietary intake was as follows (mean intake ± standard deviation), and did not significantly differ between treatment periods (all *p* > 0.05) nor from the start to the end of the trial (all *p* > 0.05): **Energy (kcal/day):** 1728.5 ± 486.4, **Carbohydrate (g/day):** 199.9 ± 64.6, **Protein (g/day):** 80.8 ± 27.1; **Fat (g/day):** 63.3 ± 28.3; **Saturated fat (g/day):** 21.8 ± 10.1; **Fibre (g/day):** 18.0 ± 8.4. Supplementary Figure 9 demonstrates in more detail the difference in fibre intake from start to end of trial at participant level.

*Metabolic profiles*

No drift was detected in QC samples. LOESS drift correction worked for all biofluids, with relative standard deviation values for faecal water, urine and serum at 7.5% (trend *p* = 0.924), 11.3% (trend *p* = 0.95), and 79.5% (trend *p* = 0.92) respectively. Alignment quality was excellent with all biofluids showing excellent peak position consistency (faecal: mean SD: 0.0046 ppm, max SD: 0.0089 ppm, all metabolites: SD < 0.01 ppm; urine: mean SD: 0.0099 ppm, max SD: 0.0174 ppm, most metabolites: SD < 0.01 ppm; serum: mean SD: 0.0031 ppm, max SD: 0.0048 ppm, all metabolites: SD < 0.01 ppm). There was no change in correlations before and after alignment due to high initial correlations and consistent peak positions.

No sequence nor phase effect was observed. PERMANOVA analysis validated the effectiveness of the crossover design with adequate washout periods (R² ≈ 0.006–0.024, *p* = 1.000). The intra-class correlation (ICC) estimated from the mixed-effects model was 0.485, closely matching with the within-subject correlation (r = 0.5) used in the power calculation. This indicates the cross-over design performed as expected. In per-protocol analyses, PERMANOVA showed no significant effect of treatment (R² = 0.0012, *p* = 0.938) or treatment × time interaction (R² = 0.0078, *p* = 0.444).

**Supplementary Figures with captions**

Supplementary Figure 1: Schematic outlining patient flow throughout PRInCE-UC, a randomised, double-blind placebo-controlled crossover clinical trial of 2’-fucosyllactose in mild-moderate ulcerative colitis (Created in BioRender. Kennedy, J., 2026)

Supplementary Figure 2: Stacked bar charts of faecal genus composition derived from 16S rRNA gene sequencing in the per-protocol population at four timepoints: pre- and post- maltodextrin (MDX) placebo, and pre- and post- 2’-fucosyllactose (2’-FL). Left: mean relative abundance (%); top 20 genera by mean abundance are shown individually, with remaining genera combined as “Other”. Right: mean absolute abundance scaled to total bacterial load using participant-matched FISH flow-cytometry counts (log₁₀ cells/mL).

Supplementary Figure 3: Horizontal bar chart of functional guild abundance differences between 2’-fucosyllactose (2’-FL) and placebo (MDX) at post-treatment in the per-protocol cohort. Guilds were defined *a priori* from genus-to-function mapping and aggregated.

Supplementary Figure 4: Mean within-person change in acetate (post − baseline) by biofluid (faecal, serum, urine) and treatment period (maltodextrin, MDX, placebo *vs* 2’-fucosyllactose, 2’-FL). Error bars represent standard error of the mean.

Supplementary Figure 5: Mean within-person change in butyrate (post − baseline) by biofluid (faecal, serum, urine) and treatment period (maltodextrin, MDX, placebo *vs* 2’-fucosyllactose, 2’-FL). Error bars represent standard error of the mean. Serum butyrate was not quantifiable and is omitted.

Supplementary Figure 6: Spearman correlation heatmap between faecal genus relative abundances and faecal NMR metabolites in the intention-to-treat (ITT) population. Each cell shows the correlation coefficient for one genus-metabolite pair across all ITT faecal samples with paired metabolomic and 16S data. The 30 most variable genera are shown on the x-axis. Metabolites on the y-axis include short-chain fatty acids, tricarboxylic acid cycle intermediates, amino acids, choline metabolites, and other quantified faecal metabolites. Colour scale: Spearman ρ (blue = negative, red = positive). Circle size: −log₁₀(nominal *p*-value) for correlations with *p* < 0.05 (larger circles = smaller *p* value).

Supplementary Figure 7: Scatter plot of within-person changes in urinary butyrate (post − baseline) during the 2’-fucosyllactose (2′-FL) period in the per-protocol (PP) population *vs* change in Simple Clinical Colitis Activity Index (SCCAI). Negative SCCAI change indicates clinical improvement. Solid line = linear regression with 95% confidence band. Exploratory change-on-change analysis; survives FDR within the urine PP correlation family.

Supplementary Figure 8: Receiver operating characteristic curve for baseline age predicting clinical response to 2’-fucosyllactose (2′-FL) in the per-protocol cohort. Response defined as ≥2-point reduction in Simple Clinical Colitis Activity Index during the 2′-FL period. Higher age predicted response (direction: older = more likely responder). Dashed diagonal = no discrimination.

Supplementary Figure 9: Volcano plot of genus-level faecal microbiota at baseline before 2′-FL, comparing participants aged ≥50 years vs <50 years; y-axis: −log₁₀ p-value (Wilcoxon rank-sum). Red points: *p* < 0.05, dashed lines mark significance thresholds. Taxa associated with older age at baseline overlap with taxa linked to 2′-FL response in age-stratified analyses.

Supplementary Figure 10: Bland-Altman plot assessing agreement between baseline and week 14 estimates of daily dietary fibre intake (g/day) derived from Nutritics food diaries in the intention-to-treat population. The x-axis shows the mean intake at the two time points; the y-axis shows the paired difference (week 14 − baseline). The red dashed line indicates the mean bias (−0.27 g/day); blue dashed lines denote the 95% limits of agreement (−14.6 to 14.1 g/day).

**References for Supplementary Material**

1. Wallner G, Amann R, Beisker W. Optimizing fluorescent *in situ* hybridization with rRNA-targeted oligonucleotide probes for flow cytometric identification of microorganisms. **Cytometry** 1993;14(2):136–43.

2. Amann RI, Binder BJ, Olson RJ, Chisholm SW, Devereux R, Stahl DA. Combination of 16S rRNA-targeted oligonucleotide probes with flow cytometry for analyzing mixed microbial populations. **Appl Env Microbiol** 1990;56(6):1919–25.

3. Daims H, Brühl A, Amann R, Schleifer KH, Wagner M. The domain-specific probe EUB338 is insufficient for the detection of all bacteria: development and evaluation of a more comprehensive probe set. **Syst Appl Microbiol** 1999;22(3):434–44.
